# Determining the Authenticity of Ghanaian Honeys Using Stable Isotope Ratio Analysis (SIRA)

**DOI:** 10.3390/molecules31142401

**Published:** 2026-07-08

**Authors:** Lebene Kpattah, Zala Sel, Marjeta Mencin, Dennis Kpakpo Adotey, Nives Ogrinc

**Affiliations:** 1 National Nuclear Research Institute, Ghana Atomic Energy Commission, Accra P.O. Box LG 80, Ghana; lebene.kpattah@gaec.gov.gh (L.K.); dennis.adotey@gaec.gov.gh (D.K.A.); 2 School of Nuclear and Allied Sciences, College of Basic and Applied Sciences, University of Ghana, Accra P.O. Box LG 67, Ghana; 3 Department of Environmental Sciences, Jožef Stefan Institute, Jamova 39, 1000 Ljubljana, Slovenia; zala.sel@ijs.si (Z.S.); marjeta.mencin@ijs.si (M.M.); 4 Research and Innovation Center Pomurje, Jožef Stefan Institute, Lendavska Ulica 5a, 9000 Murska Sobota, Slovenia

**Keywords:** honey, authenticity, stable isotopes, EA-IRMS, Ghana, food fraud, C4 sugar adulteration, PCA

## Abstract

Honey is a high-value food product that is vulnerable to adulteration with exogenous sugars, posing challenges for food authenticity and consumer protection. This study applied Stable Isotope Ratio Analysis (SIRA) to assess the authenticity of honey collected from three major honey-producing regions of Ghana (Volta, Bono and Bono East). A total of 28 honey samples were analysed by elemental analysis–isotope ratio mass spectrometry (EA-IRMS) to obtain carbon (*δ*^13^C), nitrogen (*δ*^15^N) and sulphur (*δ*^34^S) isotope composition. Honey authenticity was evaluated according to AOAC Official Method 998.12 by comparing *δ*^13^C values of bulk honey and the corresponding protein fraction. The *δ*^15^N and *δ*^34^S values in honey protein were used to investigate environmental and regional variability. Samples without detectable C4 adulteration exhibited *δ*^13^C_protein_ values consistent with C3 floral sources, whereas several samples showed Δ*δ*^13^C values more negative than −1.0‰, indicating the presence of C4-derived sugars above the AOAC adulteration threshold. Calculated C4 sugar contents ranged from 8 to 12% in moderately adulterated samples to as high as 78–79% in severely adulterated samples, confirming substantial dilution with C4 sugars. Nitrogen and sulphur isotope ratios provide additional information on environmental and regional variability among the sampled regions. Principal Component Analysis revealed that the first two principal components (PC1 and PC2) accounted for 83.8% of the total variance 83.8% of the total variance and showed separation between samples with detectable C4 adulteration and those without, while highlighting regional isotopic differences. These results demonstrate that stable isotope analysis is an effective tool for detecting C4 sugar adulteration in honey and that the combined use of carbon, nitrogen and sulphur isotopes can provide additional information on environmental and regional variability. These findings provide preliminary isotopic data on honey collected from three major honey-producing regions of Ghana and support the application of the stable isotope approach for honey authenticity assessment and quality control.

## 1. Introduction

Honey is a natural sweet substance produced by honeybees from floral nectar and is valued worldwide for its nutritional, economic, and cultural importance. In Ghana, honey production contributes to rural livelihoods and local food systems and represents an increasingly important agricultural activity. The country encompasses diverse ecological zones ranging from coastal savannahs to forests and transitional ecosystems, supporting a wide variety of nectar-producing plant species and honey production systems [1,2,3]. This diversity contributes to variability in honey composition and provides an interesting framework for authenticity and provenance studies [4]. The country has also obtained certification for honey export to the European Union, highlighting the growing economic importance of the sector [5].

Honey is among the most frequently adulterated food commodities worldwide [6]. Recent large-scale investigations, such as the European Commission’s “From the Hives” operation, found that almost 46% of honey samples imported into the EU were flagged for non-compliance, mainly due to the presence of added sugar syrups [7]. Honey adulteration can be broadly classified into direct and indirect adulteration. Direct adulteration involves the addition of exogenous sugar syrups to honey after harvest to increase product volume and economic profit. Common adulterants include high-fructose corn syrup, cane sugar syrup, invert sugar syrup, rice syrup, beet syrup, and other industrial sweeteners that closely resemble the natural carbohydrate composition of honey, making their detection challenging using conventional analytical methods. Indirect adulteration occurs when honeybee colonies are fed sugar syrups during nectar collection or honey production, resulting in the incorporation of these sugars into the honey by the bees themselves. Among the various adulterants, syrups derived from C4 plants, particularly high-fructose corn syrup and cane sugar syrup, have historically been the most frequently encountered due to their low cost, widespread availability, and sugar profiles similar to those of authentic honey.

Because these syrups are so difficult to detect through basic sensory or chemical screening, stable carbon isotope ratio analysis provides a robust method for detecting such adulteration. This approach is based on differences in photosynthetic pathways between C3 plants (typical nectar sources) and C4 plants (common sugar sources). C3 plants generally exhibit *δ*^13^C values between −33‰ and −22‰, whereas C4 plants range from −16‰ to −10‰ [8,9]. For detection, the AOAC 998.12 (EA-IRMS) method is considered the “gold standard” for identifying C4 sugars [10]. This method relies on the principle that honey protein, serving as the internal standard, accurately reflects the true C3 floral source. If C4 sugars are introduced, the overall honey values will shift to more positive (or less negative) ratios.

However, detecting adulteration with C3 plant sugars (e.g., rice or beet syrups) remains challenging because their isotopic signatures overlap with those of genuine honey. Detection of these more sophisticated forms of adulteration generally requires complementary analytical approaches [11], including liquid chromatography–isotope ratio mass spectrometry (LC-IRMS) [12,13], high-resolution mass spectrometry (LC-HRMS) [14,15], nuclear magnetic resonance (NMR) spectroscopy [16], or oligosaccharide profiling, which provide improved sensitivity for identifying C3 sugar additions and other complex adulteration practices.

Beyond carbon isotopes, nitrogen (*δ*^15^N) and sulphur (*δ*^34^S) isotope ratios measured in honey proteins provide additional information related to environmental and geographical variability. Nitrogen isotope ratios may reflect differences in nitrogen cycling, soil characteristics, and agricultural practices, whereas geological substrates, environmental sulphur sources, and marine sulphate deposition influence sulphur isotopes [4]. Previous studies have demonstrated that multi-isotope approaches combining carbon, nitrogen, and sulphur isotopes can improve the characterisation of honey production systems and support geographical differentiation. In particular, sulphur isotopes have been identified as one of the most informative tracers for distinguishing honey from different environmental settings, while nitrogen isotopes provide complementary information on production environments [17,18,19].

Although honey production is well established in Ghana, limited information is available regarding the authenticity status and isotopic characteristics of honey produced in different regions of the country. In particular, studies evaluating the combined application of carbon, nitrogen, and sulphur isotopes for authenticity assessment and regional characterisation of Ghanaian honey remain scarce.

The objectives of this study were (i) to assess honey authenticity using Stable Isotope Ratio Analysis according to AOAC Official Method 998.12, (ii) to identify potential C4 sugar adulteration in honey samples collected from major honey-producing regions of Ghana, and (iii) to evaluate regional isotopic variability using carbon, nitrogen, and sulphur isotope ratios. The study does not seek to provide definitive proof of overall honey authenticity or geographical provenance but rather to evaluate consistency with established isotope-based authenticity criteria and explore the potential of multi-isotope approaches for honey characterisation. To our knowledge, this is the first study to evaluate honey authenticity in Ghana using a combined stable-isotope approach incorporating carbon, nitrogen and sulphur isotope ratios. In addition to applying AOAC Official Method 998.12 for C4 sugar adulteration detection, the study provides an initial regional isotopic dataset for Ghanaian honey while exploring the potential of multi-isotope analysis for authenticity assessment and characterisation of regional variability.

## 2. Results and Discussion

### 2.1. Detection of C4 Sugar Adulteration Using Stable Isotopes

The authenticity of honey samples was evaluated using Stable Isotope Ratio Analysis (SIRA) via elemental analysis–isotope ratio mass spectrometry (EA-IRMS). This method detects adulteration by comparing the carbon isotopic composition of bulk honey with that of its protein fraction.

According to AOAC Official Method 998.12 [10,20] and Codex Alimentarius standards [21], the authenticity thresholds for pure honey typically range from −28‰ to −22.5‰ (*δ*^13^C value of pure honey, C3 plants). The difference (Δ*δ*^13^C) between the protein and the bulk honey (*δ*^13^C_protein_ − *δ*^13^C_bulk_) should be ≥−1.0‰. For an adulterated honey, a difference of more than −1.0‰ indicates the presence of C4 sugars. A sample with a calculated percentage sugar (% Sugar) value above 7% (>7%) is generally considered a non-pure or adulterated sample.

The percentage of C4 sugar was calculated using the isotopic difference between the protein and bulk honey fractions according to:

%*C*4 *Sugar* = (*δ*^13^*C_protein_* − *δ*^13^*C_bulk_*)/(*δ*^13^*C_protein_* − (−9.7)) × 100 (1)

A reference value of −9.7‰ was used to represent the typical *δ*^13^C signature of C4 plant sugars (e.g., sugarcane or maise). This calculation assumes that the protein fraction reflects the original C3 botanical source, while deviations in the bulk honey reflect the addition of exogenous C4 sugars.

#### 2.1.1. Regional Pattern of Adulteration

##### Volta Region

The isotopic results from the Volta region (Table 1) reveal a clear distinction between the production and retail stages. Based on AOAC Official Method 998.12, samples were classified as AOAC-compliant, moderately adulterated, or severely adulterated according to their Δ*δ*^13^C values and estimated C4 sugar content.

Of the eight samples analysed, three (37.5%) were found to be adulterated. All farm-derived samples (HV1–HV4) complied with the AOAC 998.12 criteria and showed no evidence of detectable C4 sugar adulteration. They exhibit δ^13^C_bulk_ values within the expected C3 range (−26.0‰ to −25.0‰) and Δ*δ*^13^C values between −0.8‰ and 0.3‰. These values are within the expected isotopic range of C3 reported for honey derived predominantly from C3 floral sources [4,17,18,19].

In contrast, three of the four retail samples (HV5, HV6, HV8) showed strong evidence of adulteration. These samples exhibited significantly high *δ*^13^C_bulk_ values (−14.4‰ to −13.5‰), while *δ*^13^C_protein_ remained within the C3 range (~−26‰), resulting in highly negative Δ*δ*^13^C values (−11.91‰ to −12.44‰). The corresponding C4 sugar contents (72–76%) indicate extensive dilution with C4 sugars.

These results indicate that adulteration was detected primarily among retail samples from the Volta region. The absence of detectable C4 sugar adulteration in farm-derived samples and its presence in several retail samples suggest that adulteration may occur after production during processing, distribution, or retail handling. This pattern aligns with findings in Brazil, China, and Turkey [22,23], where higher rates of adulteration were reported in urban markets compared to farm-level samples. However, independent verification of supply-chain practices would be required to determine the exact stage at which adulteration occurred. Therefore, the observed distribution of adulterated samples should be interpreted as evidence of potential supply-chain vulnerabilities rather than definitive proof of where adulteration was introduced.

##### Bono East and Bono Regions

The results (Table 2) reveal a diverse range of authenticity throughout the regions. Moreover, a notable portion of the samples meets international purity standards. For instance, samples like HB5 and HB8 show almost no difference between the honey and its protein content, indicating no detectable evidence of C4 sugar adulteration according to AOAC 998.12.

The isotopic results from the Bono East and Bono regions reveal two distinct levels of adulteration: moderate and severe C4 sugar addition.

Moderately adulterated samples (HB1, HB2, HB6, HB9, HB10 and HB12) exhibited Δ*δ*^13^C values below −1.0‰, ranging from −1.1‰ to −1.8‰, corresponding to C4 sugar contents of approximately 8–12%. These values indicate partial dilution of honey with C4-derived sugars such as cane sugar or corn syrup. Despite this modification, the δ^13^Cbulk values of these samples remain relatively close to the expected C3 range, suggesting that a substantial proportion of the original honey matrix remains present in the mixture.

Samples HB13 and HB14 represent cases of severe adulteration, with *δ*^13^C_bulk_ values around −13‰ and calculated C4 sugar contents of 78–79%. These values are characteristic of pure C4 plant sugars and indicate that these samples are composed predominantly of exogenous syrups with a substantially reduced proportion of the original honey matrix.

The presence of both moderate and severe adulteration highlights a broad spectrum of product integrity within the Bono regions. Adulteration was detected in samples collected directly from producers and in retail samples, indicating that adulterated products may be present at multiple points along the supply chain. This result contrasts with the Volta region, where farm samples without detectable C4 adulteration were observed, and adulteration was confined to retail outlets.

The occurrence of adulteration at multiple stages of the supply chain reflects structural challenges within Ghana’s honey sector. As noted by different studies [24], honey production is largely decentralised and dominated by small-scale producers, making consistent quality control difficult. This lack of quality control may facilitate both intentional dilution and inconsistent handling practices.

The observed pattern is consistent with findings from Benjamin et al. [25], who reported varying levels of adulteration in Ghanaian markets, including evidence of both partial and extensive syrup addition. Their study suggests that honey adulteration is more prevalent in market samples than at the point of production. However, the present results indicate that this distinction is less clear in the Bono regions.

The isotopic patterns observed in the Bono and Volta regions suggest differences in the distribution of adulterated samples across the supply chain. However, without independent traceability data, these findings should be interpreted as indications of potential vulnerabilities rather than confirmation of the precise stage at which adulteration occurred. The observed adulteration patterns may also reflect economic incentives stemming from the high market value of honey relative to industrial sugar syrups, particularly in supply chains involving multiple actors. Such economic drivers have been identified as important factors contributing to food fraud and honey adulteration worldwide [6,26,27].

Overall, the coexistence of AOAC-compliant, moderately adulterated and severely adulterated samples within the same region emphasises the complexity of honey authenticity in Ghana. These findings highlight the need for routine isotopic monitoring and stronger regulatory oversight to address both subtle and severe forms of adulteration across the supply chain.

### 2.2. Regional Comparison: Carbon (δ^13^C_protein_), Nitrogen (δ^15^N_protein_) and Sulphur (δ^34^S_protein_)

While carbon isotopes provide robust evidence for detecting C4 sugar adulteration, nitrogen (*δ*^15^N) and sulphur (*δ*^34^S) isotopes in honey protein offer complementary insights into the environmental and geographical origin of honey. These isotopes reflect local soil conditions, agricultural practices and regional geochemistry and have therefore been widely investigated as potential tracers of honey origin [4,18,19].

**Figure 1:**
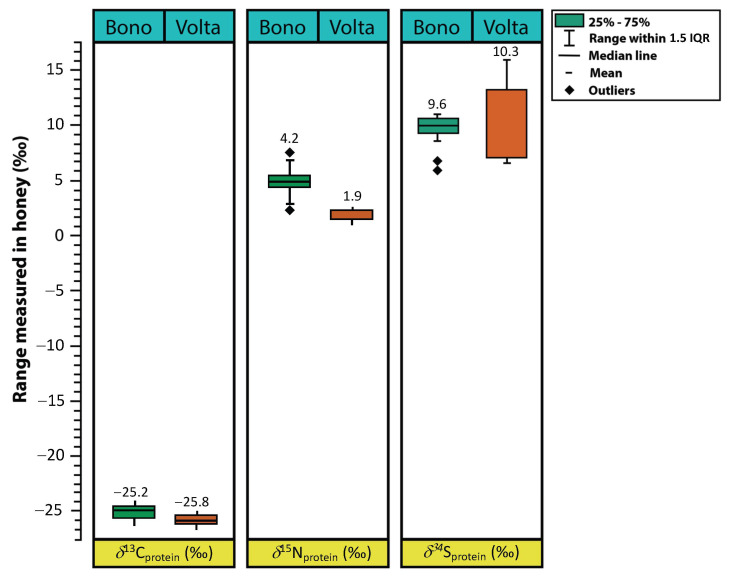
Box-and-whisker plots showing the distribution of *δ*^13^C_protein_, *δ*^15^N_protein_ and *δ*^34^S_protein_ values in honey samples collected from the Volta, Bono and Bono East regions of Ghana.

The isotopic composition of honey proteins differed among the Volta, Bono and Bono East regions, reflecting both environmental variability and potential geographical influences (Figure 1).

The *δ*^13^C_protein_ values showed relatively limited variation among regions, indicating that the protein fraction retained the original botanical carbon signature. Similar observations have been reported in other studies [4,17,18,19], which found that *δ*^13^C_protein_ values were not significantly influenced by botanical origin and remained relatively stable across different production areas. The authors reported *δ*^13^C_protein_ values ranging from approximately −26.8 to −24.4‰, comparable to those observed in the present study. These findings support the use of the protein fraction as a stable-isotopic reference for assessing honey authenticity.

More pronounced differences were observed for *δ*^15^N_protein_ values. Samples from the Volta region exhibited relatively low *δ*^15^N_protein_ values (1.3–2.1‰), whereas samples from the Bono and Bono East regions generally showed higher values (3.7–6.6‰). Such differences may reflect variations in nitrogen cycling, soil characteristics and agricultural management practices. Similar regional variability has been reported in previous studies, in which nitrogen isotope ratios have been shown to contribute to regional characterisation in multi-isotope studies. However, *δ*^15^N_protein_ may also be influenced by botanical origin and should therefore be interpreted together with other isotopic markers. Comparable observations were reported by Kropf et al. [17] and Bonini et al. [19], who found that *δ*^15^N_protein_ values reflected environmental and agricultural differences among honey production areas.

Sulphur isotopes provided additional information on regional variability. AOAC-compliant samples from the Volta region exhibited a broader *δ*^34^S_protein_ range (9.3–16.0‰) than samples from the Bono and Bono East regions (8.5–11.0‰). Sulphur isotopes are strongly influenced by local geology and environmental factors such as marine sulphate deposition. The elevated δ^34^S_protein_ values observed in some Volta samples may reflect coastal influences, commonly referred to as the “sea-spray effect” [28]. Similar findings were reported by Bonini et al. [19], who identified *δ*^34^S_protein_ as one of the most informative variables for geographical discrimination of honey and demonstrated significant differences between coastal and inland production regions.

Together, the nitrogen and sulphur isotope data complement the carbon isotope results by providing additional information on environmental and geographical variability. The combined use of carbon, nitrogen and sulphur isotopes therefore strengthens honey characterisation and supports the application of multi-isotope approaches for authenticity and provenance studies.

### 2.3. Principal Component Analysis (PCA)

**Figure 2:**
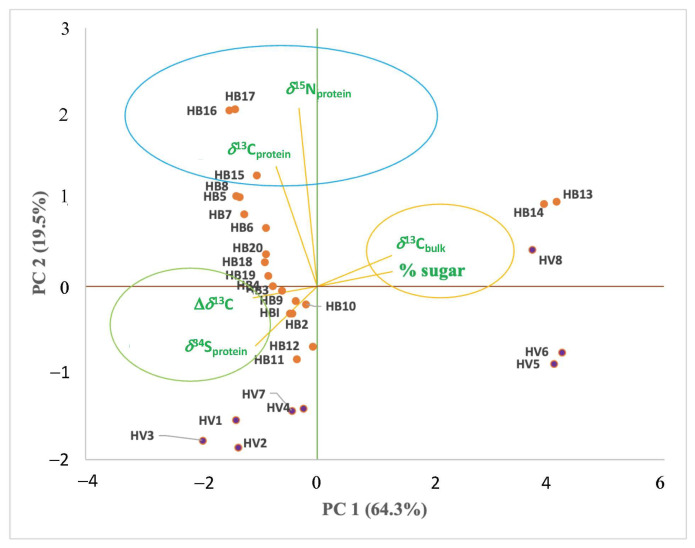
Principal Component Analysis (PCA) of honey samples based on *δ*^13^C_bulk_, *δ*^13^C_protein_, *δ*^15^N_protein_ and *δ*^34^S_protein_ values. The first two principal components explained 83.8% of total variance and revealed separation between honey samples from the Volta and Bono regions.

Principal Component Analysis (PCA) was performed using *δ*^13^C_bulk_, *δ*^13^C_protein_, *δ*^15^N_protein_, *δ*^34^S_protein_, Δ*δ*^13^C, and calculated C4 sugar content (%C4 sugar) to evaluate patterns among honey samples from the Volta and Bono/Bono East regions (Figure 2). The first two principal components explained 83.8% of the total variance, with PC1 accounting for 64.3% and PC2 for 19.5%.

Samples from the Volta and Bono/Bono East regions tended to cluster differently along PC1. However, the observed patterns should be interpreted cautiously, given the limited sample size and lack of independent verification. Most Volta samples were positioned on the negative side of PC2, whereas Bono/Bono East samples clustered predominantly on the positive side. These patterns suggest differences in isotopic composition among the sampled regions and indicate the potential of multi-isotope analysis to support the characterisation of honey from different production environments. However, further studies involving larger sample sets, seasonal replication, and independently authenticated reference samples are needed to confirm these observations.

The loading plot showed that *δ*^13^C_bulk_ and calculated C4 sugar content were strongly and positively associated with PC1, while Δ*δ*^13^C was negatively associated with this component. Samples exhibiting high positive PC1 scores corresponded primarily to adulterated samples (HV5, HV6, HV8, HB13 and HB14), characterised by higher *δ*^13^C_bulk_ values and elevated C4 sugar contents. These variables, therefore, represent the major drivers of adulteration-related discrimination among samples.

In contrast, *δ*^13^C_protein_ and *δ*^15^N_protein_ contributed mainly to the negative side of PC1 and positive values of PC2, where most AOAC-compliant HB samples were located. The relatively stable *δ*^13^C_protein_ values confirm that the protein fraction retained its original botanical signature despite dilution of the bulk honey. The higher *δ*^15^N _protein_ values observed in Bono/Bono East samples may reflect differences in local environmental conditions, nitrogen cycling, soil characteristics, and agricultural practices compared with the other sampled regions.

The *δ*^34^S _protein_ variable was associated with variation along PC2 and provided additional information on environmental and geographical variability. The broader range of *δ*^34^S _protein_ values observed in Volta samples compared with those in Bono/Bono East samples may be related to differences in local geology and potential marine influences.

Overall, the PCA results suggest that the combination of carbon, nitrogen and sulphur isotope ratios can assist in distinguishing AOAC-compliant and adulterated honey samples while providing information on regional variability. The strongest discrimination was achieved through variables directly related to C4 sugar adulteration (*δ*^13^C_bulk_, Δ*δ*^13^C and %C4 sugar), whereas *δ*^15^N_protein_ and *δ*^34^S_protein_ values showed regional variability that may reflect differences among the sampled production areas.

### 2.4. Study Limitations

The isotopic evidence presented in this study clearly identifies samples exhibiting C4 sugar adulteration, as defined by AOAC Official Method 998.12. However, several limitations should be acknowledged. Samples collected directly from beekeepers and producers were used as reference materials representative of local production systems, but were not independently authenticated through external traceability systems or forensic verification procedures. No independent verification methods, such as melissopalynological analysis, elemental profiling, LC-IRMS, NMR spectroscopy, or DNA-based approaches, were applied to confirm botanical or geographical origin. Consequently, conclusions regarding origin should be regarded as indicative rather than definitive.

In addition, the applied methodology, based on AOAC Official Method 998.12 and EA-IRMS analysis of bulk honey and its protein fraction, is specifically designed to detect adulteration involving C4 plant sugars and cannot exclude adulteration involving C3 sugar sources such as rice or beet syrups. While the observed isotopic patterns provide valuable information on regional differences and potential supply-chain vulnerabilities, independent verification of production practices and product traceability would be required to determine the stage at which adulteration occurred.

Future studies incorporating larger sample sets, multiple harvesting seasons and independent botanical verification would further strengthen regional interpretation. The present study should be regarded as an exploratory assessment of isotope-based indicators of honey integrity rather than a definitive authentication or provenance study. Independent verification of sample authenticity, larger sample numbers, seasonal replication and complementary analytical approaches would be required before establishing robust regional isotopic reference frameworks for Ghanaian honey.

## 3. Materials and Methods

### 3.1. Study Area and Collection of Honey Samples

#### 3.1.1. Study Area

**Figure 3:**
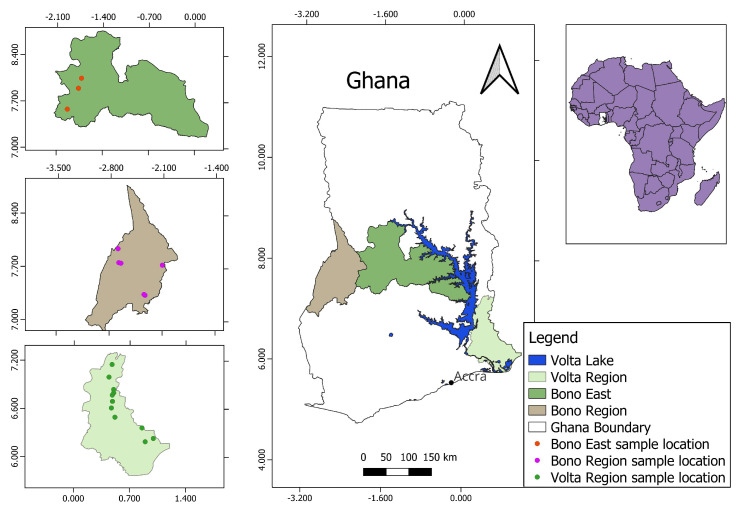
Map of Ghana showing the three sampling regions with sampling locations.

Honey samples were collected from three major honey-producing regions of Ghana: the Volta, Bono and Bono East regions (Figure 3). These regions were selected because they represent distinct environmental and agricultural settings that may influence honey’s isotopic composition [1,2,3]. The Volta region is characterised by coastal, forest and transitional ecosystems. In contrast, the Bono and Bono East regions are located within the forest–savannah transition zone and are dominated by agricultural activities, including cocoa, cashew and fruit production. Differences in vegetation, land use and environmental conditions among these regions provide an opportunity to evaluate regional isotopic variability and honey authenticity [29].

#### 3.1.2. Sample Collection

Honey samples were collected following previously published methodologies and recommended sampling protocols [15,30]. A total of 28 samples were collected, twenty (20) from both the Bono East and Bono regions and eight (8) from the Volta region, with samples weighing around 250 to 500 g. To effectively detect adulteration, a two-pronged sampling strategy was used: (i) Manufacturer/Apiary Samples (Producer Reference Samples) collected directly from beekeepers representative of local production systems, and (ii) Commercial Samples (Market Surveillance) gathered from a variety of retail outlets, including open markets, roadside stalls and supermarkets in bustling urban areas across the three regions. It should be noted that these beekeeper-sourced samples were used as reference materials for comparison purposes and were not independently authenticated through external traceability systems, chain-of-custody documentation, or verification of feeding practices. Consequently, they should not be interpreted as certified authentic controls in a forensic sense.

The honey samples were packed in leakproof, food-grade HDPE (High-Density Polyethylene) containers. The primary container was then placed inside a leakproof secondary bag (a heavy-duty Ziploc). Each packed sample was uniquely labelled and transported to the Department of Environmental Sciences at the Jožef Stefan Institute in Ljubljana, Slovenia, for isotopic measurement.

### 3.2. Sample Preparation

In honey, the ^13^C/^12^C ratio in the bulk sample and ^13^C/^12^C, ^15^N/^14^N, and ^34^S/^32^S ratios in honey protein were determined. The stable isotope ratio measurements were performed using isotope ratio mass spectrometry (IRMS) and expressed in the *δ* notation in ‰ according to Equation (2) [31]:

(2) δ(Ei/j)=δEi/j=RPi/j−RRefi/jRRefi/j,

where superscripts *i* and *j* denote the higher and the lower atomic mass number of element *E*, and *R_p_* and *R_Ref_* represent the ratios between the heavier and the lighter isotopes (^13^C/^12^C, ^15^N/^14^N, ^34^S/^32^S) in the sample (*P*) and reference material (*Ref*), respectively. The *δ*^13^C values are reported relative to the V-PDB (Vienna Pee Dee Belemnite) standard. *δ*^15^N and *δ*^34^S are reported relative to AIR and the V-CDT (Vienna Canyon Diablo Troilite) standard, respectively.

#### 3.2.1. Bulk Honey

For the bulk honey ^13^C/^12^C analysis, about 1.4 mg of the honey was loaded into an aluminium capsule using a microbalance XPR3DUE (Metter Toledo, Greifensee, Switzerland) with a capacity of 3.2 g. Using two pairs of ultra-fine tweezers, the tops of the tin capsules were folded over and crimped, ensuring they were air-tight.

#### 3.2.2. Protein Extraction

The protein in each honey sample was extracted according to AOAC 998.12 (Association of Official Analytical Chemists Handbook) [10]. To extract the internal protein standard, 10–12 g of each honey sample (measured using an analytical balance XPE205 (Metter Toledo, Greifensee, Switzerland); Max 220 g) was dissolved in 4 mL of Milli-Q water (Millipore, Burlington, MA, USA) in a clear 50 mL centrifuge tube. A total of 2.0 mL of sodium tungstate (10% *w*/*v*) and 2.0 mL of sulfuric acid (0.33 M) were added to precipitate the proteins [13]. The mixture was heated to 80 °C in water (Julabo 33 water bath, Julabo, Seelbach, Germany) until visible floc formed.

The tube was filled with ultrapure water, homogenised, and centrifuged (Centric 322A centrifuge, Domel, Železniki, Slovenia) for 5 min at 32,000 rpm, and the supernatant was decanted. The washing, homogenisation and centrifuging steps were repeated 10 times with 50 mL portions of ultrapure water, thoroughly decanting the supernatant each time to remove any unwanted sugars.

The resulting protein pellets were placed in an oven at 60 °C until dry. Once dry, the protein pellets are crushed completely into a fine powder using a laboratory spatula. About 2.3–2.4 mg of powdered protein was also carefully weighed into an aluminium capsule. Using two pairs of ultra-fine tweezers, the tops of the tin capsules were folded over and crimped into an air-tight seal.

### 3.3. Elemental Analysis–Isotope Ratio Mass Spectrometry (EA-IRMS)

Determination of *δ*^13^C values in bulk honey and *δ*^13^C, *δ*^15^N and *δ*^34^S values in protein isolated from honey was performed by an IsoPrime100 Vario PYRO Cube (OH/CNS Pyrolyser/Elemental Analyzer) (IsoPrime, Cheadle Hulme, UK).

For the determination of *δ*^13^C values in bulk honey, the results were normalised against international reference materials: USGS-77 with a value of δ13C = −30.71 ± 0.04‰, and IAEA-CH-6 with a value of *δ*^13^C = −10.449 ± 0.033‰. For the independent control, a reference material, USGS83, with a value of *δ*^13^C = −26.20 ± 0.08‰ was used.

For determination of *δ*^13^C, *δ*^15^N and *δ*^34^S values in protein isolated from honey, the results for carbon and nitrogen were normalised against the following international reference materials: USGS64 with values of *δ*^13^C = −40.81 ± 0.04‰ and *δ*^15^N = 1.76 ± 0.04‰; USGS88 with values of *δ*^13^C = −16.06 ± 0.07‰ and *δ*^15^N = 14.96 ± 0.16‰; and USGS89 with values of *δ*^13^C = −18.13 ± 0.11‰ and *δ*^15^N = 6.25 ± 0.12‰. For the independent control laboratory reference materials (CRP-IAEA-2013 with values of *δ*^13^C = −20.3 ± 0.09‰ and *δ*^15^N = 5.62 ± 0.19‰, and USGS42 with a value of *δ*^15^N = 8.05 ± 0.10‰) were used. The results for sulphur were normalised against the following reference materials: USGS88 with a value of *δ*^34^S = 17.10 ± 0.40‰, USGS89 with a value of *δ*^34^S = +3.86 ± 0.56‰, and USGS42 with a value of *δ*^34^S = 7.84 ± 0.25‰. For the independent control, a reference material, CRP-IAEA casein, with a value of *δ*^34^S = 4.18 ± 0.79‰ was used. All samples were analysed in triplicate. Analytical precision, determined from repeated measurements of standards and selected samples, was better than ±0.2‰ for *δ*^13^C and *δ*^15^N and ±0.5‰ for *δ*^34^S. Quality-control standards were analysed at regular intervals throughout the analytical sequence to verify instrument stability.

### 3.4. Statistical Evaluation

Descriptive statistics were calculated for all isotope data and are presented as mean ± standard deviation (SD). Differences in isotope ratios among regions were evaluated using box-and-whisker plots. Principal Component Analysis (PCA) was performed using *δ*^13^C_bulk_, *δ*^13^C_protein_, *δ*^15^N_protein_, *δ*^34^S_protein_, Δ*δ*^13^C, and calculated C4 sugar content (%C4 sugar) to explore patterns of sample grouping, regional differentiation, and adulteration status. All statistical analyses were performed using Microsoft Excel Professional Plus 2021 (Microsoft Corporation, Redmond, WA, USA) and XLSTAT 2024 (Addinsoft, Long Island, NY, USA) software.

## 4. Conclusions

This study demonstrates the effectiveness of Stable Isotope Ratio Analysis (SIRA) in assessing the authenticity of Ghanaian honey. By comparing the isotopic composition of bulk honey (*δ*^13^C_bulk_) with that of its protein fraction (*δ*^13^C_protein_), varying degrees of adulteration were successfully identified.

The results reveal that while most farm-derived samples remained authentic, a significant proportion of retail samples exhibited Δ*δ*^13^C values more negative than the internationally accepted threshold of −1.0‰, confirming the addition of C4-derived sugars such as cane sugar or high-fructose corn syrup. Moderate adulteration (≈8–12% C4 sugar) was detected in several samples, including some at the production level, indicating that adulteration is not limited to retail markets. More critically, heavily adulterated samples exhibited extreme isotopic shifts, with calculated sugar contents of up to 78–79%, suggesting that these products consist largely of exogenous syrups rather than genuine honey.

The isotopic evidence clearly demonstrates the occurrence of C4 sugar adulteration in a proportion of the analysed samples, including cases of severe adulteration. However, samples classified as authentic should be interpreted as showing no detectable C4 sugar adulteration according to the AOAC 998.12 criteria. The applied methodology cannot exclude adulteration involving C3 sugar sources or provide absolute verification of production practices and provenance.

Beyond carbon isotopes, nitrogen (*δ*^15^N_protein_) and sulphur (*δ*^34^S_protein_) analyses revealed patterns consistent with regional variability among the sampled areas. Variations in *δ*^15^N_protein_ values reflected differences in soil nitrogen cycling and agricultural practices between regions, while *δ*^34^S_protein_ values captured regional geological signatures and coastal influences.

These findings reveal distinct distributions of adulterated samples between regions. In the Volta region, adulteration was detected primarily among retail samples, whereas in the Bono region, adulterated samples were identified at both producer and retail levels. However, independent traceability investigations would be required to determine the exact point at which adulteration occurred.

Overall, this study emphasises the urgent need for routine isotopic monitoring, stricter regulatory enforcement and improved traceability systems for honey. Additionally, the results provide an initial foundation for the future development of a national multi-isotope reference database for honey authentication in Ghana.

## Figures and Tables

**Table 1:** Stable isotope results of honey from the Volta region in Ghana, together with the Δ*δ*^13^C and % of C4 sugar addition.

Source	Sample ID	*δ*^13^C_bulk_ (‰)	*δ*^13^C_protein_ (‰)	*δ*^15^N_protein_ (‰)	*δ*^34^S_protein_ (‰)	Δ*δ*^13^C (‰)	%C4 Sugar	Status
Farm	HV1	−26.0 ± 0.0	−25.7 ± 0.1	2.1 ± 0.0	13.0 ± 0.2	0.3	0	AOAC-compliant
Farm	HV2	−25.4 ± 0.1	−25.5 ± 0.1	1.3 ± 0.1	13.6 ± 0.2	−0.1	0	AOAC-compliant
Farm	HV3	−25.0 ± 0.1	−25.1 ± 0.1	1.3 ± 0.1	16.0 ± 0.1	−0.1	0	AOAC-compliant
Farm	HV4	−25.3 ± 0.1	−26.1 ± 0.1	2.1 ± 0.1	9.4 ± 0.1	−0.8	0	AOAC-compliant
Retail	HV7	−26.0 ± 0.0	−26.1 ± 0.1	2.1 ± 0.0	9.3 ± 0.1	−0.1	0	AOAC-compliant
Retail	HV5	−14.4 ± 0.0	−26.6 ± 0.1	2.3 ± 0.0	7.3 ± 0.2	−12.2	72	Severely adulterated
Retail	HV6	−13.6 ± 0.0	−26.0 ± 0.1	1.1 ± 0.1	6.5 ± 0.3	−12.4	76	Severely adulterated
Retail	HV8	−13.5 ± 0.1	−25.4 ± 0.1	2.6 ± 0.0	7.0 ± 0.2	−11.9	76	Severely adulterated

**Table 2:** Stable isotope results of honey from Bono East and Bono regions in Ghana, together with the Δ*δ*^13^C and % of C4 sugar addition.

Source	Sample ID	*δ*^13^C_bulk_ (‰)	*δ*^13^C_protein_ (‰)	*δ*^15^N_protein_ (‰)	*δ*^34^S_protein_ (‰)	Δ*δ*^13^C (‰)	%C4 Sugar	Status
Farm	HB3	−25.0 ± 0.1	−26.7 ± 0.1	4.2 ± 0.2	9.7 ± 0.0	−0.7	0	AOAC-compliant
Farm	HB4	−25.2 ± 0.1	−25.5 ± 0.1	4.0 ± 0.2	9.6 ± 0.1	−0.3	0	AOAC-compliant
Farm	HB5	−24.8 ± 0.2	−24.9 ± 0.0	5.3 ± 0.0	10.6 ± 0.1	−0.1	0	AOAC-compliant
Farm	HB7	−24.4 ± 0.2	−24.7 ± 0.1	4.6 ± 0.0	10.7 ± 0.2	−0.3	0	AOAC-compliant
Farm	HB8	−24.5 ± 0.2	−24.5 ± 0.0	4.6 ± 0.1	10.5 ± 0.1	−0.0	0	AOAC-compliant
Retail	HB11	−25.0 ± 0.0	−25.8 ± 0.0	2.6 ± 0.0	9.3 ± 0.1	−0.8	0	AOAC-compliant
Retail	HB15	−24.3 ± 0.2	−25.1 ± 0.0	6.1 ± 0.0	10.4 ± 0.1	−0.8	0	AOAC-compliant
Retail	HB16	−24.4 ± 0.3	−24.5 ± 0.0	6.6 ± 0.1	10.4 ± 0.0	−0.1	0	AOAC-compliant
Retail	HB17	−24.1 ± 0.2	−24.5 ± 0.0	6.6 ± 0.1	10.4 ± 0.1	−0.4	0	AOAC-compliant
Retail	HB18	−24.5 ± 0.1	−25.0 ± 0.0	3.8 ± 0.1	9.9 ± 0.1	−0.5	0	AOAC-compliant
Retail	HB19	−24.6 ± 0.1	−25.2 ± 0.0	3.7 ± 0.1	10.0 ± 0.1	−0.5	0	AOAC-compliant
Retail	HB20	−24.5 ± 0.1	−25.0 ± 0.0	3.8 ± 0.1	9.7 ± 0.0	−0.5	0	AOAC-compliant
Farm	HB1	−24.9 ± 0.1	−26.3 ± 0.0	4.9 ± 0.0	10.2 ± 0.2	−1.4	8	Moderately adulterated
Farm	HB2	−24.8 ± 0.1	−26.1 ± 0.0	4.8 ± 0.2	10.8 ± 0.1	−1.3	8	Moderately adulterated
Retail	HB9	−23.4 ± 0.0	−24.5 ± 0.2	1.5 ± 0.0	8.5 ± 0.0	−1.1	8	Moderately adulterated
Farm	HB6	−23.4 ± 0.2	−24.9 ± 0.1	4.5 ± 0.2	11.0 ± 0.0	−1.5	10	Moderately adulterated
Retail	HB10	−23.1 ± 0.0	−24.7 ± 0.0	1.6 ± 0.1	8.5 ± 0.1	−1.6	11	Moderately adulterated
Retail	HB12	−23.7 ± 0.1	−25.5 ± 0.0	2.5 ± 0.0	9.4 ± 0.1	−1.8	12	Moderately adulterated
Retail	HB13	−13.1 ± 0.1	−25.8 ± 0.1	4.2 ± 0.2	5.9 ± 0.1	−12.7	79	Severely adulterated
Retail	HB14	−13.3 ± 0.2	−25.8 ± 0.0	4.5 ± 0.2	6.8 ± 0.1	−12.5	78	Severely adulterated

## Data Availability

The datasets generated and analysed in this study are available from the corresponding author on reasonable request.
